# The Types of Learning Approaches Used by Engineering Students in Three Scenarios: An Adaptation of the R-SPQ-2F to China

**DOI:** 10.3389/fpsyg.2022.944588

**Published:** 2022-07-08

**Authors:** Chunyu Zhao, Haiyang Hou, Qiongying Gu

**Affiliations:** ^1^School of Standardization, China Jiliang University, Hangzhou, China; ^2^School of Science, Hangzhou Dianzi University, Hangzhou, China; ^3^Office of Academic Affairs, NingboTech University, Ningbo, China

**Keywords:** deep learning, engineering students, R-SPQ-2F, theoretical learning, experimental learning, practical learning

## Abstract

Deep learning is a type of high-level learning that has received widespread attention in research on higher education; however, learning scenarios as an important variable have been ignored to some extent in past studies. This study aimed to explore the learning state of engineering students in three learning scenarios: theoretical learning, experimental learning, and engineering practice. Samples of engineering university students in China were recruited online and offline; the students filled in the engineering Education-Study Process Questionnaire, which was revised from the R-SPQ-2F. The results of clustering analysis showed four types of learning approaches in the three scenarios: typical deep learning, typical shallow learning, deep-shallow learning, and free learning. Engineering learners in different learning scenarios tended to adopt different learning approaches and showed gender differences. Due to factors such as differences in culture and choice of learning opportunities, the deep and shallow learners demonstrated excellent learning performance, which is in sharp contrast with the “learning failure” exhibited by such students abroad.

## Introduction

In higher education, engineering degrees account for a third of the degrees globally and comprise a significant part of higher education in China. Given today’s surging wave of the Fourth Industrial Revolution, there is an urgent need for reforming engineering education, and improving the quality of engineering talent training is a top priority (Hariharasudan and Kot, 2018). However, a large volume of engineering education research studies has found that problems remain in the practice of engineering education in China, such as low student engagement (Ding, 2020), poor enthusiasm and interaction (Wang et al., 2019), varied learning motives, and a relatively low sense of mission (Zhan et al., 2016). How to improve learning quality for engineering students is one of the areas of focus for engineering college educators and administrators. Engineering learning is highly practical. Apart from classroom learning, engineering students’ experimental hours and inside–outside school practical hours account for more than 30% of their total course hours. Hence, exploring learning characteristics under different learning scenarios is of great significance for improving the learning quality experienced by engineering students.

In most studies of college student learning, deep learning has received attention because it focuses on the nature of learning and its potential meaning (Marton and Saljo, 1976; Beatie et al., 1997; Ngidi, 2014; Lake and Boyd, 2015; McLaughlin and Durrant, 2016; Zakariya et al., 2020; Takase and Yoshida, 2021; Tannoubi et al., 2022). It is proposed in the Bologna Declaration that “Successful learning necessarily include deep learning.” The Horizon Report in 2017 even stated that deep learning is the key direction for promoting the development and reform of higher education in the next 5 years or more. In the “Opinions on Deepening the Reform of Undergraduate Education and Teaching and Comprehensively Improving Talent Training Quality in China” (Professor of Engineering, 2019, No. 6) published by the Ministry of Education in 2019, the sentence “We should strive to establish five kinds of “golden courses” with high-order, innovative and challenging characteristics” coincides with the requirements of deep learning.

Deep learning is being applied in many areas of higher education. Studies have also found that a deep learning approach exerts a positive impact on learning outcomes (Hassall and Joyce, 2001; Snelgrove and Slater, 2010), and also positively affects personality and intelligence development (Chamorro-Premuzic and Furnham, 2008; Nelson Laird et al., 2014). However, there are few deep learning studies on engineering students, and most of the current research on deep learning focuses on one learning scenario. It is also worth paying attention to whether there are differences in learning paths in different learning scenarios.

In view of this, this study adopts the classic deep learning dichotomy model (Marton and Saljo, 1976) as the analysis framework to investigate the state of deep learning for engineering students in the contexts of three learning types, theoretical learning, experimental learning, and engineering practice. It provides a picture of engineering students in China engaging in deep learning and analyzing multiple dimensions. Finally, it identifies learning characteristics so as to provide a reference for a better understanding of the learning state of engineering students in China and to improve learning quality.

## Literature Review

In 1976, Marton and Saljo, Swedish educational psychologists and professors at the University of Gothenburg, argued that learners’ processing can be divided into deep learning and surface learning. As soon as the concept of deep learning came into being, it aroused the interest of many international scholars. Among the many researchers interested in deep learning, the dichotomy model from Biggs, an Australian scholar, has enjoyed popularity due to its simplicity and accessibility. Biggs believed that surface learning mainly refers to learning through repeated practice or memory based on external interest or motive, with the aim of avoiding failure or passing exams (Biggs, 1988). In contrast, deep learning is based on internal motive or interest (Xie et al., 2022), which emphasizes an in-depth understanding of learning content and shows that students know what they have already mastered and what they need to work hard at and are willing to expend more effort on (Chiou et al., 2012). Biggs also developed a research tool for deep learning for the first time from the perspective of assessment – the Study Process Questionnaire (SPQ) for college students (Biggs, 1987a). The original construct of the SPQ was composed of three different elements – surface, deep, and achieving learning – and each element contained two measurement sub-scales of motivation and strategy (Biggs, 1987b; Wong et al., 1996). However, many studies of the content structure of the SPQ suggest that surface and deep learning as a two-factor solution is more appropriate for explaining individual learning (Watkins and Akande, 1994; Kember and Leung, 1998; Jeong et al., 2019). Biggs optimized the investigation of the original learning process from the two dimensions of motive and strategy (Biggs et al., 2001). The revised version, called the R-SPQ-2F, consists of a short 20-question questionnaire with a five-point Likert scale. It is very simple to employ and utilize, and it has been adapted into many languages, such as Spanish (Justicia et al., 2008), Turkish (Önder and Besoluk, 2010), Japanese (Fryer et al., 2012), Dutch (Stes et al., 2013), Chinese (Xie, 2014), and Arabic (Shaik et al., 2017). The R-SPQ-2F has also been adapted to study learning in many fields, such as biology (Biggs, 1987b), innovation education (Zhang, 2020), medicine (Liang et al., 2018), nursing (Alsayed et al., 2021), and others. The validity of the R-SPQ-2F has been confirmed by the above studies. Thus, with the R-SPQ-2F, the current research can target extending our understanding of the learning characteristics of engineering students.

Based on the two-dimensional model framework of deep learning, relevant research on the deep learning of engineering students in China is based on the two dimensions of motive and strategy. Deep learning motive has been confirmed as one of the key factors constraining engineering students from solving problems; a higher-level deep learning motive leads to higher teaching satisfaction and a stronger problem-solving ability (Lizzio et al., 2002). However, the overall learning motive of engineering students varies, and they have been found to have a relatively low sense of mission (Zhan et al., 2016). The active participation frequency of engineering undergraduates is significantly lower than that of students overall, while differences occur in terms of different genders, school types, and grades; male students have been found to have a higher level of problem-solving ability than female students (Ding, 2020). Thus, in relevant studies on the learning of engineering students in China, deep learning motive and strategy are slightly involved, but there are relatively few comprehensive studies on deep learning. The two-dimensional model of deep learning developed by Biggs provides structural simplicity and accessibility, and empirical studies have been carried out in many countries (Mauricio et al., 2018; Shigeo and Rieko, 2018), confirming that it has high reliability and validity and can be used as a reference for relevant studies on the deep learning of engineering students.

## Research Tools and Data Sources

### Research Tools

In this study, the Revised Study Process Questionnaire (R-SPQ-2F) developed by Biggs is used as a reference for variable measurement and operationalization. R-SPQ-2F contains four dimensions: deep learning motive (DM) and deep learning strategy (DS), and surface learning motive (SM) and surface learning strategy (SS), with a total of 20 questions. Among them, the two dimensions of deep learning motive and deep learning strategy are combined to form deep learning behavior, while the two dimensions of surface learning motive and surface learning strategy are combined to form surface learning behavior. Each test question in R-SPQ-2F contains four answer items, which are, respectively, A: I never or rarely do this, B: I sometimes do this, C: I do this about half the time, D: I often do this; 1 to 4 points are given, respectively, for each of A to D. The CFI of the four sub-dimensions in the questionnaire are all greater than 0.9, and the Cronbach’s Alpha is between 0.57 and 0.72 (Biggs et al., 2001).

The Engineering Students’ Learning Experience questionnaire compiled by this research institute was revised based on R-SPQ-2F, and it consists of two parts: the first part is a background survey, which aims to collect students’ identity attribute information, including their gender, grade, school type, and academic performance. The second part is a questionnaire on deep learning. Based on the differences in the learning content and learning styles of engineering students, deep learning was measured according to the three scenarios of theoretical learning, experimental learning, and engineering practice. Firstly, after translating and revising the description of R-SPQ-2F according to Chinese language habits, 20 engineering undergraduates were invited for a site test, and suggestions for revision of the test questions were proposed from the two aspects of content understanding and fitness of description. The test questions were revised according to the suggestions, and some test questions were removed or merged; 14 test questions were retained in each scenario. In the second round, 10 experts in the field of pedagogy were consulted on the rationality of the test question descriptions to ensure the best possible accuracy of the description of the test questions. Table 1 shows the theoretical learning module as an example and a specific test is presented.

**TABLE 1:** Deep learning (theoretical learning module) test questions for college students.

Dimension	Code	Content
Deep learning motive	DM1	I find that at times theoretical studying gives me a feeling of deep personal satisfaction.
	DM2	I feel that virtually any theoretical topic can be highly interesting once I get into it.
	DM3	I find that studying academic topics can at times be as exciting as a good novel or movie.
	DM4	I work hard at my studies because I find the material interesting.
Deep learning strategy	DS1	I find that I have to do enough work on a theoretical topic so that I can form my own conclusions before I am satisfied.
	DS2	I find most new theoretical topics interesting and often spend extra time trying to obtain more information about them.
	DS3	I test myself on important theoretical topics until I understand them completely.
	DS4	I spend a lot of my free time finding out more about interesting theoretical topics which have been discussed in different classes.
Shallow learning motive	SM1	My aim is to pass the theoretical course while doing as little work as possible.
	SM2	I do not find my theoretical course very interesting, so I keep my work to the minimum.
	SM4	I find I can get by in most theoretical courses assessments by memorizing key sections rather than trying to understand them.
Shallow learning strategy	SS1	I only study seriously what’s given out in class or in the theoretical course outlines.
	SS2	I learn some things by rote, going over and over them until I know them by heart even if I do not understand them.
	SS3	I generally restrict my study to what is specifically set as I think it is unnecessary to do anything extra.

A total of 408 engineering students from six universities in Zhejiang province were tested to confirm the reliability and validity of the questionnaire. There were 240 male students and 168 female students, consisting of 81 freshmen, 127 sophomores, 113 juniors, 69 seniors, and 18 postgraduates. The Cronbach’s Alpha was greater than 0.7, achieving the requirements of measurement indexes (shown in Table 2).

**TABLE 2:** Cronbach’s Alpha of each dimension.

Scenarios	Dimension	Cronbach’s Alpha
Theoretical learning	DM	0.909
	DS	0.859
	SM	0.804
	SS	0.782
Experimental learning	DM	0.940
	DS	0.886
	SM	0.793
	SS	0.828
Engineering practice	DM	0.950
	DS	0.900
	SM	0.887
	SS	0.903

### Data Sources

The formal survey was administered from May 2020 to April 2021, and a total of 3,779 copies of the valid questionnaire were collected in two batches, online and offline. Among them, 2,695 engineering students accounted for 71.32% of the total, 811 students from other majors accounted for 21.46%, and 273 students did not report their majors. In the engineering samples, there were 1,688 male students, accounting for 62.63% of the total, and 1,006 female students, accounting for 37.33%, while one student did not report his/her gender. There were 980 freshmen, accounting for 36.36% of the total; 575 sophomores, accounting for 21.34%; 360 juniors, accounting for 13.36%; 368 seniors, accounting for 13.65%; 409 postgraduates, accounting for 15.18%, and three students did not report their grade. In this study, engineering students are taken as the object for analysis.

## Research Findings

### Clustering and Naming of Learning Types: Different Learning Groups of Engineering Students Are Demarcated by Four Learning Types

During the learning process, there are strong individual differences in the purpose of learning, understanding, and way of accomplishing learning tasks. Individuals with a knowledge comprehension worldview try to figure out and grasp the true meaning of something, and often adopt deep processing, while individuals with the worldview of knowledge accumulation turn to surface processing with repetition and memory as strategies (Prosser et al., 2000), and present deep or surface processing characteristics in both the dimensions of learning motive and learning strategy. So how do current engineering students establish their learning style? Is there a relatively stable group of students with specific learning preferences and what are the characteristics in terms of the composition of these student groups? K-means is adopted in this study to cluster engineering students in the three learning scenarios of theoretical learning, experimental learning, and engineering training. The study attempts to explore the specific group of engineering students who engage in deep learning and to determine their specific learning motives and strategic preferences on this basis.

**FIGURE 1:**
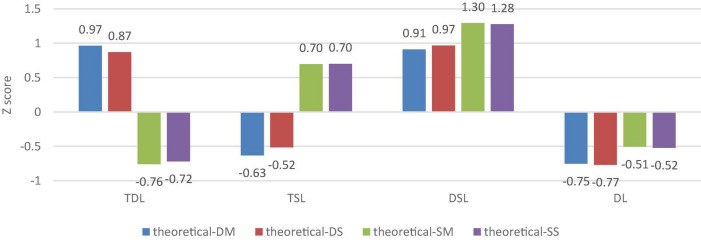
Learning scores of engineering students of different learning types in theoretical learning scenarios.

The results show that students can be stably clustered into four groups in any situation. Taking the theoretical study, for example (see Figure 1), the first group of students gains a higher than average score for the two dimensions of deep learning motive and strategy, while it obtains a lower than average score for surface learning motive and strategy. These students usually stimulate a deep learning motive and adopt a deep learning strategy no matter what the curriculum or teachers, so they are perceived as the good students working hard in everyone’s eyes, accounting for about 23.20% of the total number of students. The second group of students scores higher than average on surface motive and strategy, but lower than average on deep motive and strategy, which is the method of using simple memory for learning in all courses, and this group of students accounts for about 22.55% of the total. The third group scores higher than average on deep learning motive and strategy and also gains a higher than average score for surface learning motive and strategy; these students may stimulate different learning motives and adopt different learning strategies based on particular theoretical courses or teachers. For example, in a higher mathematics course, some engineering students may be satisfied using a formula to pass the exam instead of understanding the mathematical principles behind the formula and theorem; moreover, some engineering students may struggle to understand the principle behind the formula in a probability and statistics course. This group accounts for about 17.40% of the total number of students. The fourth group of students gains a lower than average score for both deep learning motive and strategy and surface learning motive and strategy. They are neither expected to pass the exams of theoretical courses nor do they pursue deep mastery. They muddle along and belong to the type who do not learn; this group accounts for about 36.86% of the total.

Students in the scenarios of experimental learning and engineering training are, respectively, clustered with the same method, enabling four groups of students with similar characteristics to be obtained. The clustering results are like those from Lv (2018), and there are only differences in proportions due to the variability of samples and learning scenarios. This indicates that it is a relatively stable technique to demarcate students into four learning groups or four deep learning styles based on different motives and strategies of deep learning. We, respectively, name these four groups of students as typical deep learning type, typical surface learning type, deep and surface shared learning type, and dissociative learning type.

#### Typical Deep Learners (TDL)

In terms of the scores gained, these learners have a higher score for deep learning and a lower score for surface learning. In the dimension of motive, such learners are driven by the internal learning motive and regard learning as fun. As for learning strategies, deep processing and learning strategies are applied. In the three scenarios of theoretical, experimental, and engineering training, this group of students, respectively, accounts for 23.20, 23.99, and 24.57% of the total, showing no significant difference. Namely, among the current population of engineering students, this group of students accounts for nearly a quarter.

#### Typical Surface Learners (TSL)

In the scores obtained, these learners have a higher score for surface learning and a lower score for deep learning. They have the motive to avoid failure; namely, they learn to avoid failing instead of really mastering knowledge and they lack real interest in learning. In terms of the strategic dimension, they allocate “just enough” time for learning task accomplishment and are unwilling to input more energy into learning; they fail to experience fun during learning. In the three scenarios of theoretical, experimental, and engineering training, this group of students, respectively, accounts for 22.55, 14.90, and 24.57% of the total.

#### Deep and Surface Shared Learners (DSL)

These learners have higher scores for both deep learning and surface learning. This result is unexpected and paradoxical. The students sometimes work hard due to interesting lessons and sometimes make an effort to pass exams. Sometimes they adopt learning strategies oriented at mastery and comprehension, and sometimes they turn to memory strategies based on memorization and retelling. Such a learning approach was first discovered by Meyer et al. (1990) and Entwistle et al. (1991). Chinese scholar Lv Linhai also discovered such a group in the deep learning of general education courses, accounting for 15–20% of learners (Lv, 2018). In the present study, in the three scenarios of theoretical, experimental, and engineering training, this group of students, respectively, accounts for 17.40, 23.55, and 17.53% of the total.

#### Dissociative Learners (DL)

These learners have lower scores for both deep learning and surface learning. In terms of motive, these students study not for the purpose of passing exams or being interested in learning since they are not driven by an intrinsic motive. They probably do not want to study, or they “consume time” in school for their parents or due to other external factors. They are unwilling to input more time at the strategic level and even may not input energy into homework. In the three scenarios of theoretical, experimental, and engineering training, this group of students, respectively, accounts for 36.86, 37.56, and 33.33% of the total. Such a high quantity of dissociative students shows the worrying situation of engineering students in China and shows that there is still a long way to go in the reform of engineering education.

All the scores of different dimensions were obtained from the three learning scenarios. The proportion of students involved in the four learning types is shown in Table 3.

**TABLE 3:** Proportion of engineering students with the four learning types.

TDL	DSL	DL	TSL
21.86%	13.58%	29.87%	34.69%

### Cultural Differences in Specific Learning Types: “Disconnected Students” Involved in Deep and Surface Shared Learning Type May Not Be “Disconnected”

Biggs, the developer of SPQ, through numerous empirical studies, has reached the basic consensus that deep learning leads to better learning outcomes, while surface learning often results in learning failure (Snelgrove and Slater, 2010). However, the cluster analysis in the present study revealed a special group of engineering students who adopt both a deep learning method, characterized by mastery, and a surface learning method, characterized by surface recitation and retelling, showing the contradiction and inconsistency of learning motive and strategy. Prosser et al. (2000) provided a concise description of these learners, calling them “disconnected students” who adopted what he termed a method of “integrative learning.” Prosser stated that such students present a “disconnected” phenomenon of contradiction, incoherence, and inconsistency in their recognition of learning scenarios, motive drive, strategy selection, and the results they achieve, and such students tend to be the group with the worst academic performance (Prosser et al., 2000). In our study, the number of learners with a deep and surface shared learning type accounted for about 13.58% of the total, so have these engineering students also suffered a failure in learning?

This study measures students’ consistent learning motive and learning strategy, and general learning outcomes are taken to predict their learning success. Specifically, students’ learning outcomes were predicted according to their grade ranking in the class last year, their subjective perception of learning, and how they like their subject (Table 4). It is found that for engineering students in China, the students with a deep and surface shared learning type do not “disconnect,” but show excellent learning outcomes. They do not behave differently from deep learners in the above three aspects, and their performance is significantly better than that of students with a dissociative learning type and surface learning type.

**TABLE 4:** Differences in learning performance, sense of gain, and professional satisfaction among engineering students of different types (mean ± standard deviation).

Comparative indices	TDL	DSL	DL	TSL	F	Significance level
Grade ranking in the class last year	2.30 ± 1.175	2.48 ± 1.246	2.60 ± 1.270	2.77 ± 1.310	14.467**	0.00
Subjective perception of learning	3.82 ± 0.726	3.57 ± 0.943	3.15 ± 0.842	3.23 ± 0.837	80.575**	0.00
How they like their subject	4.05 ± 0.946	3.66 ± 1.131	3.20 ± 1.024	3.27 ± 1.051	83.044**	0.00

*The two asterisks represent P < 0.01.*

Why deep and surface shared learners had been proven to fail in western experiments, while they gained good results in this study? Professor Lv Linhai at Nanjing University once explained this phenomenon as follows: “Chinese students’ surface learning is accompanied with the moral factor of efforts.” He believed that the surface learning measured through the SPQ questionnaire among Chinese students is not mechanical “memory” or “recitation,” but memory with an understanding of the meaning (Lv, 2018). As Zhu Xi, a famous neo-Confucianism in the Song Dynasty, emphasized in *the Essentials of Reading*, “repeated and familiar reading” and “recitation and memorization” are the basic elements for subsequent understanding.

We consider that this finding may also be explained from the perspective of both Eastern and Western students’ choice of learning content. Western colleges and universities widely adopt a complete credit system. Thus, the occurrence of the deep and surface shared learning type can be attributed to meta-cognitive deficiency. In contrast, course supply in Chinese colleges and universities is relatively unified, and a high proportion of courses are compulsory credits, meaning that there is limited scope for students’ selection and free choice of courses. Thus, in the learning scenario of Chinese college students, some students do not “accept without question” the teaching that they receive, and instead evaluate it with critical eyes and determine their own learning strategies. That is to say, Chinese students apply a “preliminary value judgment” to the learning experience they are about to receive, which is a manifestation of learning meta-cognition and also a sign of students’ initiative in the selection of their learning experience. Learning meta-cognition is closely associated with clarity in students’ life planning; namely, the clearer students’ learning career planning is, the easier it is for them to consciously choose learning content and monitor their learning effort and dedication. This could explain why students involved in deep and surface shared learning can achieve better learning outcomes.

### Differences in Different Types of Scenarios: Learning Mode Selection in Experimental Learning Scenarios Presents Different Characteristics

Science is aimed at discovering new knowledge, engineering aims to create new things, humanities aim to discover the self, and the social sciences aim to optimize society. Different disciplines have their specific values and purposes. The engineering educational paradigm is the basic framework for the cultivation of engineering talent, which is shown at the level of school education. In conformity with differences in learning content, learning patterns, and interaction patterns between teachers and students, there are theoretical learning scenarios based on one-to-many teaching, which is mainly located in the classroom; an experimental learning scenario based on the combination of one-to-many and one-to-one teaching guidance, which is mainly located in laboratories; and an engineering practice scenario based on a one-to-one or one-to-few mode, which is mainly located in engineering practice settings. The above three learning scenarios can also be understood as three different curriculum systems. Studies have shown that when students enter the learning environment of a real and objective classroom (course), their subjective “course perception” of the environment, to the greatest extent, will affect whether they adopt the “deep learning” strategy (Prosser and Trigwell, 1999). Do engineering students abide by their own learning styles in different types of course surroundings? We have, respectively, compared the proportion of engineering students who adopt the four types of learning style, finding that in the experimental learning scenario, the proportion of students involved in the surface learning type is significantly lower than that of the theoretical and engineering training scenarios, and the proportion decreases by 7.65 and 9.67%, respectively. The shaded module in the spider web diagram in Figure 3 significantly deviates to the right, indicating that in the experimental scenario, since basic operations such as manually operating instruments for measurement and conducting experiments are in need, adopting surface learning based on strategies of memory and retelling may not help engineering students accomplish learning tasks smoothly. On the other hand, we have found that in the experimental learning scenario, the proportion of learners applying the deep and surface shared learning type is much higher than in the other two scenarios, and the proportion is, respectively, 6.15 and 6.01% higher than that in the theoretical and engineering training scenarios. Thus, it can be speculated that when students are in the experimental learning scenario, more students choose whether to conduct deep learning in accordance with experiment type, difficulty, and method.

### Differences in Varied Learning Types: Male Engineering Students Are More Selective About Their Learning Experience

**FIGURE 2:**
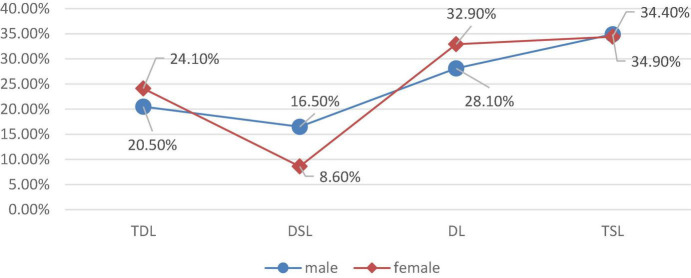
Proportion of male and female engineering students with different deep learning styles.

Gender is an important influencing factor of individual attributes in college students’ choice of learning style. Due to the influence of social expectations, family, and school education, students of different genders show differences in learning behavior performance. We applied Pearson’s chi-squared test to analyze the difference in the distribution of deep learning types between male and female engineering students. The results show that the chi-square value is 41.00, and the concomitant probability is 0.000. There is a huge difference in the distribution of deep learning types between male and female students. Specifically, this is shown by the fact that the proportion of male learners involved in the deep and surface shared learning is about 16.5%, which is far higher than the 8.6% of female learners involved in this learning type, while the group of dissociative female learners accounts for 32.90% of the total, 4.8% higher than that of male students (see Figure 2).

**FIGURE 3:**
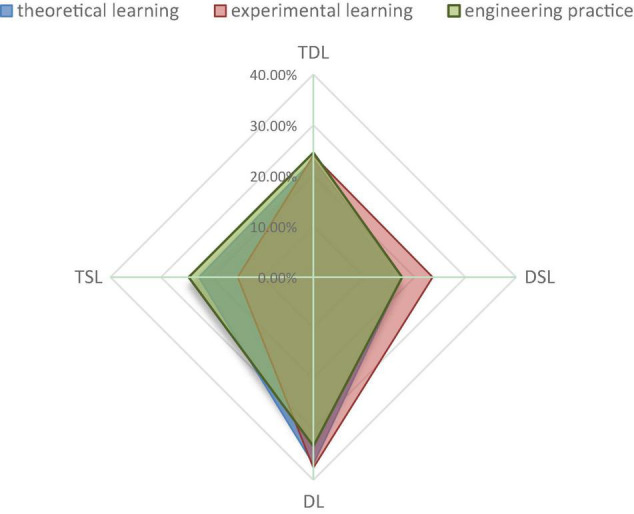
Proportion of different learning styles in different learning course scenarios.

In the context of the Chinese educational environment, learners involved in deep and surface shared learning show stronger meta-cognition and self-monitoring, and we are more willing to describe this type of learners as demonstrating “more flexibility” in their choice of learning style, and these learners also attain a relatively excellent learning performance. Male students are more flexible in their choice of learning style, while female students are relatively rigid and consistent in their choice of learning style. However, there is also a “risk” in this finding; that is, in the educational environment, compared with female students, male engineering students may have a clearer learning purpose and perceive more learning tasks as having meaning and value, thus mobilizing higher-order thinking, engaging in learning, and striving to solve problems. However, they may also be more likely to “muddle along” and have an attitude of “long live the 60 points” if they face learning tasks, which they do not perceive as having value. Meanwhile, the proportions of female deep and dissociative learners are both 4% higher than that of the male learners, indicating that females show great differentiation during learning, and that some females work harder than males, but some abandon and dissociate themselves from learning.

### Grade Differences in Different Types: The Choice of Learning Types Varies With Grade Changes

**FIGURE 4:**
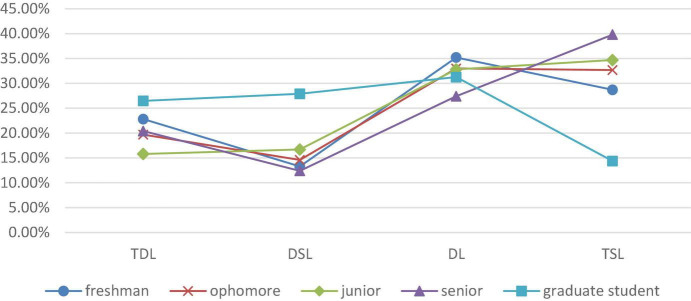
Proportion of different types of learners at undergraduate and graduate levels.

The proportion of graduate students involved in deep learning and deep and surface shared learning is much higher than that of undergraduate students. In the dimension of grade, the Pearson’s chi-squared test shows that there is no significant difference in the distribution of engineering undergraduates involved in deep learning in different grades. However, there are significant differences in the distribution of deep learning types between graduate students and undergraduates, indicating that the proportion of graduate students involved in deep learning, and surface shared learning and deep learning, is significantly higher than that of undergraduates, while the proportion of surface learners is significantly lower than that of undergraduates (see Figure 4). The second-stage analysis of undergraduate and graduate students shows that postgraduate students have clearer learning goals and are more inclined to choose the deep learning strategy that is oriented toward high-order thinking and intrinsic motives. It may be associated with the smaller class sizes and personalized training for postgraduates, but it also suggests that compared with undergraduates, postgraduates have a more internalized and interest-oriented learning motive and are more inclined to implement the deep learning strategy.

**FIGURE 5:**
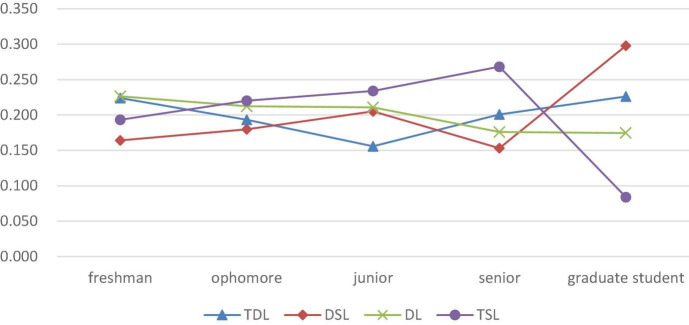
Proportion of engineering students in different grades with different deep learning styles.

There are multiple differences in the selection of learning types for undergraduates in different grades. Based on grade distribution, further analysis of the proportion of different types of learners (Figure 5) shows that the proportion of deep learners corresponds to a V-shaped distribution with grade changes, while the proportion of students engaged in deep learning in the junior year is the lowest. The proportion of learners involved in the deep and surface shared learning shows a wave of rising after a fall–rise pattern, while the proportion of dissociative learners decreases with increasing grade. The proportion of surface learners corresponds to an inverted V shape, which shows a constant rise during the undergraduate stage but suffers a sharp decline during the postgraduate stage.

Based on the above analysis, we seem to have found a “phenomenon of the junior year” in the selection of deep learning styles applied by engineering students; that is, the junior year is the key node where many students switch to a deep learning style. After this year, there is an abrupt rise in the number of students involved in deep learning, while there is a huge decline in the number of students involved in deep and surface shared learning, as well as dissociative learning. Students seem to come to a sudden “wake-up” moment when the junior year comes. From the freshman year to the junior year, the proportion of deep learners decreases with increasing grade, and the reason for this may lie in the fact that a part of deep learners in junior grade focus their interest on some goals with abandoning others, which fails to arouse interest or shows little short-term value for their own development. Instead, they change into learners engaged in deep and surface shared learning. Some of them may totally abandon a deep learning style but adopt a surface learning style due to difficulty of learning and lack of interest in learning. The junior year is a turning point where students become more mature. Stress, such as from employment and postgraduate entrance exams, urges some students to re-choose their learning behavior style to adapt to the upcoming senior year and employment challenges.

In view of the above analysis, it is suggested that junior courses should be improved in the following two aspects: first, because college students’ learning objectives are becoming clearer, to increase the freedom of senior college students in course learning, more opportunities for choosing courses to meet students’ needs should be provided. Second, the examination mode and difficulty of senior courses should be strengthened. The junior grade has entered into the phase of professional learning. In China, the syllabus, course teaching, and course evaluation are usually performed by one teacher. For those worried about the students’ comments on teaching, some teachers may lower the requirement of the system thinking and comprehensive ability evaluation; instead, students can pass the exam by remembering relevant conceptual knowledge, which may lead to more students applying a shallow learning approach. This also reflects the existing problems existing in China’s engineering education.

## Conclusion

We found that engineering students in China could be divided into four learning groups: typical deep learning type, typical surface learning type, deep and surface shared learning type, as well as dissociative learning type. Groups who select the learning style of experimental learning scenarios differ from those who select the theoretical and engineering training scenarios. This is an interesting finding which is helpful for learning research in engineering experimental scenarios. Grade difference analysis found a “junior year” phenomenon exists in the learning state of engineering undergraduates; that is, the junior year becomes the key node of switching toward a deep learning style. Chinese and Western learners who are involved in deep and surface share learning show differences in learning performance, and the study contends that students’ choice of learning content may be an important factor of influence.

Some limitations still existed. First, due to the lack of large-scale student interviews, the inferred results may not be accurate enough. In addition, this study only discussed three engineering student learning scenarios (theoretical study, experimental study, and practice study). In fact, further engineering scenarios exist, such as metalworking practice and electronic practice; the two belong to practical scenarios, but due to the different learning content and form they may refer to different learning characteristics.

Subsequent studies may focus on the influencing factors behind the deep learning approach adopted by engineering students, including teacher factors, curriculum factors, and personality factors. Findings could be obtained through individual interviews, observation, and field research, among other methods. The subsequent research could also focus on more detailed engineering learning scenarios and study the learning strategy selection of engineering students in the segmented scenario, which would be helpful to show the learning characteristics of engineering students in a more comprehensive and detailed way.

## Data Availability Statement

The original contributions presented in this study are included in the article/supplementary material, further inquiries can be directed to the corresponding author.

## Author Contributions

CZ and HH: conceptualization, formal analysis, and data curation. CZ and QG: methodology. CZ: writing – original draft preparation and project administration. HH: writing – review and editing. All authors have read and agreed to the published version of the manuscript.

## Conflict of Interest

The authors declare that the research was conducted in the absence of any commercial or financial relationships that could be construed as a potential conflict of interest.

## Publisher’s Note

All claims expressed in this article are solely those of the authors and do not necessarily represent those of their affiliated organizations, or those of the publisher, the editors and the reviewers. Any product that may be evaluated in this article, or claim that may be made by its manufacturer, is not guaranteed or endorsed by the publisher.
